# A Multiple Attention Convolutional Neural Networks for Diesel Engine Fault Diagnosis

**DOI:** 10.3390/s24092708

**Published:** 2024-04-24

**Authors:** Xiao Yang, Fengrong Bi, Jiangang Cheng, Daijie Tang, Pengfei Shen, Xiaoyang Bi

**Affiliations:** 1 State Key Laboratory of Engines, Tianjin University, Tianjin 300350, China; yangxiao@tju.edu.cn (X.Y.); fr_bi@tju.edu.cn (F.B.); jiangangcheng@tju.edu.cn (J.C.); tjutangdaijie@tju.edu.cn (D.T.); shenpengfei@tju.edu.cn (P.S.); 2 State Key Laboratory of Reliability and Intelligence Electrical Equipment, Hebei University of Technology, Tianjin 300130, China

**Keywords:** diesel engine, end-to-end fault diagnosis, machine learning, attention mechanism, self-attention mechanism

## Abstract

Fault diagnosis can improve the safety and reliability of diesel engines. An end-to-end method based on a multi-attention convolutional neural network (MACNN) is proposed for accurate and efficient diesel engine fault diagnosis. By optimizing the arrangement and kernel size of the channel and spatial attention modules, the feature extraction capability is improved, and an improved convolutional block attention module (ICBAM) is obtained. Vibration signal features are acquired using a feature extraction model alternating between the convolutional neural network (CNN) and ICBAM. The feature map is recombined to reconstruct the sequence order information. Next, the self-attention mechanism (SAM) is applied to learn the recombined sequence features directly. A Swish activation function is introduced to solve “Dead ReLU” and improve the accuracy. A dynamic learning rate curve is designed to improve the convergence ability of the model. The diesel engine fault simulation experiment is carried out to simulate three kinds of fault types (abnormal valve clearance, abnormal rail pressure, and insufficient fuel supply), and each kind of fault varies in different degrees. The comparison results show that the accuracy of MACNN on the eight-class fault dataset at different speeds is more than 97%. The testing time of the MACNN is much less than the machine running time (for one work cycle). Therefore, the proposed end-to-end fault diagnosis method has a good application prospect.

## 1. Introduction

The diesel engine has been widely employed in construction machinery, ships, nuclear power, and other fields for high thermal efficiency, immense output power, and extended service life. Many complex components of the engine are prone to failure due to their prolonged operation in high temperature, high pressure, and severe vibration environments [1]. When critical components fail, it can lead to downtime, financial loss, and even life-threatening issues [2,3]. Therefore, it is significant to carry out research on engine fault diagnosis.

The fault diagnosis based on vibration signal has become a hot research topic for simple measurement, high accuracy, and non-disassembly [4]. Traditional signal processing-based methods usually require manual feature extraction, which requires strong expert knowledge. This method has two downsides: Firstly, artificial involvement leads to uncertainty of recognition results. For example, Barai [5] and Lu [6] used empirical mode decomposition (EMD) and wavelet transform to extract fault features in vibration for identification, respectively. The choice of wavelet basis functions, EMD components, and classifiers greatly affect the diagnosis [7]. Secondly, the sensitivity of artificial features varies greatly between faults, resulting in low generalizability. For example, Zhao [8] and Ke [9] used multi-channel signal entropy and multi-scale bidirectional diversity entropy to characterize the degree of faults, respectively. The results show that the applicability of features is different for various types of faults. Manual feature selection is inefficient and difficult.

The end-to-end fault diagnosis expects to use raw time-domain data as input, complete feature extraction, and classification through self-learning. Represented by deep learning, end-to-end methods have been widely used in fault diagnosis [10]. Habbouche et al. used a convolutional neural network (CNN) to classify the features extracted by variational mode decomposition [11]. The effectiveness of this method is still affected by artificial features. CNN has excellent advantages in image feature extraction. Ribeiro [12] and Wang [13] et al. used methods such as short-time Fourier transform to convert vibration signals into two-dimensional pictures, which were classified by CNN. These methods are complicated, and there is a risk of information loss in the process of dimension transformation. And, dimension transformation methods have poor generalizability. Zhao et al. proposed a CNN-based adaptive inter- and intra-class fault diagnosis method for variable operating condition gears [14]. This method efficiently implements end-to-end fault diagnosis. Du et al. used a one-dimensional convolutional neural network (1DCNN) to process vibration signals of automobile engines to achieve fault diagnosis and classification [15]. Zhao et al. proposed a multi-branch convolutional neural network with an integrated cross-entropy to identify six diesel engine faults [16].

The CNN has strong local feature extraction capabilities but has limitations because it does not utilize the sequence order information of the time-domain data. The accuracy of end-to-end fault diagnosis directly used for engine vibration is not high. Recurrent neural network (RNN) has structural advantages in temporal data processing. Huang et al. used long short-term memory networks (LSTM) to diagnose high-speed train bogie faults [17]. Qin et al. proposed a multi-scale CNN-LSTM neural network with a residual-CNN denoising module for anti-noise diesel engine misfire diagnosis [18]. Ouyang et al. proposed a new bi-directional gated recurrent unit (BiGRU) to diagnose faults in blast furnaces [19]. Zhi et al. implemented wavelet denoising combined with the CNN-LSTM for fault diagnosis of harmonic reducers, achieving better results than CNN and LSTM [20]. As mentioned above, RNN-like methods are better than CNNs for classifying temporal data. However, the RNN typically factors computation along the input and output symbol positions, aligning the positions to steps in computation time, resulting in low efficiency, which limits its application in fault diagnosis. In 2016, Zhou et al. completed the relation classification task by introducing the self-attention mechanism (SAM) into LSTM [21]. In 2017, Vaswani et al. extensively used multi-head SAM to learn text representation and then proposed the famous transformer model [22]. Since then, transformers have been widely used in natural language processing. Compared to CNN and LSTM, SAM has a stronger temporal feature learning capability. Liu et al. constructed a prediction model of the exhaust gas temperature of the marine diesel engine based on attention-LSTM [23]. However, SAM has an ordinary feature extraction ability for raw time domain data, so it is necessary to combine it with other feature extraction networks to design the model. Using CNN-like networks for feature extraction and then using SAM for learning is a better idea. However, preserving the temporal features of the original data in CNN is also a problem.

The convolutional block attention module (CBAM) [24] is proposed to enhance the feature map representation capability of the model and to selectively focus on important information. The CBAM uses spatial attention and channel attention to fully utilize the spatial and channel information of the feature map. Guo et al. proposed an end-to-end fault diagnosis method based on attention CNN and BiLSTM, in which CBAM redistributes the weights between different feature dimensions and enhances the model’s focus on important features [25]. Yao et al. considered the effect of the location of spatial and channel attention additions on the effectiveness of pavement crack detection [26]. Yan et al. incorporated the convolutional block attention module and the improved residual module into the convolutional variational autoencoder for fault diagnosis tasks [27]. Song et al. proposed a multi-source information fusion meta-learning network with CBAM for bearing fault diagnosis under a limited dataset [28]. The effect of implementing CBAM largely depends on its parameter settings, such as the configuration of spatial and channel attention mechanisms. Spatial attention is usually implemented through a convolutional layer, and the size of the convolutional kernel of this layer affects how local features are aggregated. Larger convolution kernels may capture broader spatial relationships, while smaller convolution kernels pay more attention to detailed features. Small convolution kernels pay more attention to detailed features and abnormal patterns in small areas, which is very useful for capturing small changes or early subtle fault signals. Large convolution kernels help the model understand the working status of the entire device or the overall failure mode. However, the influence of the arrangement of channel and spatial attention, as well as the kernel size, on the effectiveness of fault diagnosis has been little discussed.

In this paper, an end-to-end fault diagnosis method based on multiple attention convolutional neural networks (MACNN) is proposed. The main contributions are as follows:

(1) The paper improves the arrangement of channel attention and spatial attention in the CBAM method while optimizing the kernel size.
(2) The feature map obtained after feature extraction is sequentially recombined to preserve the temporal features of the vibration signal. The recombined feature maps are recognized using SAM.
(3) A Swish activation function is introduced to suppress “Dead ReLU”, and the dynamic learning rate curve is designed to improve the convergence efficiency.

This paper is organized as follows: Section 1 introduces the research background and significance. Section 2 shows the fundamental algorithm theories. Section 3 includes the engine fault simulation experiment and data processing. The MACNN is proposed in Section 4. The results of diesel engine fault diagnosis based on MACNN are analyzed in Section 5. Conclusions are given in Section 6.

## 2. Background Theories

### 2.1. Convolutional Neural Networks

Convolutional neural networks are the most widely used deep learning algorithms in the field of computer vision. Their essential components mainly include the input, convolutional, pooling, fully connected, and output layers. The convolutional layer and the pooling layer appear alternately to extract features and reduce dimensions [29].

The convolutional layer uses multiple convolutional kernels to convolute with the local area of input data, and each convolutional kernel shares the weights in the convolutional process. The specific process of convolution is shown in (1).

(1) $$y_{k}^{l}=\sum w_{k}^{l}x_{j}^{l}+b_{k}^{l}$$

where $x_{j}^{l}$ is the *j*-th input block of the *l*-th layer and $w_{k}^{l}$ and $b_{k}^{l}$ are the weights and biases of the *k*-th convolutional kernel of the *l*-th layer.

The activation function can strengthen the nonlinear expression ability of the model. The ReLU function is a widely used activation function [30], and its expression is shown in (2).

(2) $$f(x)=\left\{\begin{array}{c} x\;x>0\;\\ 0\;x\leq 0\; \end{array}\right.$$

The ReLU activation function has a gradient of 0 when the input is negative, which will cause the neurons to be unable to update, resulting in the “Dead ReLU” problem. The solution to this problem will be explained later.

To further reduce the risk of over-fitting and reduce the dimensions of data, the pooling layer is often used for down-sampling after the convolutional layer. Usually, there are two ways of average pooling and maximum pooling. To fully extract the impact characteristics in the vibration signal and filter out part of the noise, the maximum pooling operation is adopted in this paper, as shown in (3).

(3) $$p_{k}^{l+1}(j)=\underset{(j-1)w+1\leq t\leq jw}{\max}\left\{a_{k}^{l}(t)\right\}$$

where $a_{k}^{l}(t)$ is the activation value of the *t*-th neuron in the *k*-th feature plane in the *l*-th layer, w is the width of the pooling area, and $p_{k}^{l+1}(j)$ is the corresponding value of the *l* + 1-th layer.

### 2.2. Convolutional Block Attention Module

**Figure 1:**
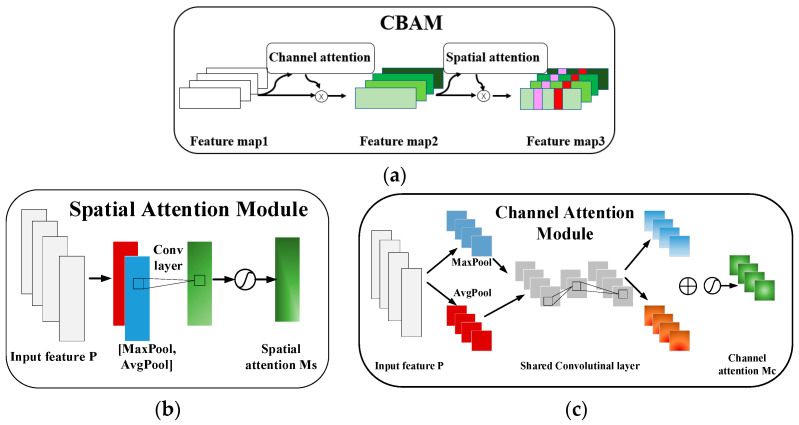
Attention modules. (**a**) The architecture of original CBAM; (**b**) channel attention module; (**c**) spatial attention module.

CBAM combines the ideas of CNNs and attention mechanisms, with the main advantage of adaptively learning important features for a specific task. Attention mechanisms enable the model to focus on the most relevant features for the task at hand. By dynamically adjusting the importance of different features, attention mechanisms help extract discriminative information from the input data, leading to improved performance. In addition, attention mechanisms allow the model to selectively attend to specific parts of the input data. This selective feature extraction enables the model to focus on relevant information while ignoring irrelevant or noisy features, leading to more robust representations. There are channel attention modules and spatial attention modules in CBAM. The structure of CBAM, channel attention module, and spatial attention module are shown in Figure 1.

The channel attention focuses on the connections across the channel in the feature map. The channel attention module $M_{C}(\cdot )$ is calculated by (4).

(4) $$M_{C}(P)=\sigma (Conv2(Conv1(P_{\mathrm{avg}}^{c}))+Conv2(Conv1(P_{\max}^{c})))$$

where $P_{\mathrm{avg}}^{c}\in {\mathbb{R}}^{C\times 1\times 1}$ and $P_{\max}^{c}\in {\mathbb{R}}^{C\times 1\times 1}$ are the global average pooling feature and global maximum pooling feature across the spatial axis. *Conv*1 and *Conv*2 share the same parameters for both inputs and they are connected by the activation function, whereby the convolutional kernel size in *Conv*1 and *Conv*2 will be discussed later, and σ is the activation function.

The spatial attention $M_{S}(\cdot )$ focuses on the connections across the spatial regions, calculated by (5).

(5) $$M_{s}(P)=\sigma (Conv([P_{\mathrm{avg}}^{s},P_{\max}^{s}]))$$

where $P_{\mathrm{avg}}^{s}\in {\mathbb{R}}^{1\times H\times W}$ and $P_{\max}^{s}\in {\mathbb{R}}^{1\times H\times W}$ are the global average pooling feature and global maximum pooling feature across the channel axis, $[P_{\mathrm{avg}}^{s},P_{\max}^{s}]\in {\mathbb{R}}^{2\times H\times W}$ is spliced by $P_{\mathrm{avg}}^{s}\in {\mathbb{R}}^{1\times H\times W}$ and $P_{\max}^{s}\in {\mathbb{R}}^{1\times H\times W}$, whereby the convolutional kernel size will be discussed later, and σ is the activation function.

### 2.3. Self-Attention Mechanism

SAM is a special form of attentional mechanism that improves the ability to model correlations between different positions in a sequence [24]. The attention weight is calculated based on (6).

(6) $$W=softmax(\alpha^{T}(Tanh(X^{\prime })))$$

where α is a trained parameter vector, and the initial value is given by random initialization at the beginning of training and then adjusted by the gradient descent algorithm. $W=\left\{w_{1},w_{2}\cdot \cdot \cdot w_{l^{\prime }}\right\}$ is the weight learned from the recombined sequence feature $X^{\prime }$. The larger the $w_{l^{\prime }}$ is, the more important the information is to the classification decision.

Finally, the result is obtained by multiplying the attention weight and sequence features, as shown in Equation (7).

(7) $$y=Tanh(X^{\prime }W^{T})$$

where *y* is the result of SAM.

## 3. Data Preparation

Failure data of the diesel engine was obtained through simulation experiments. The engine fault simulation bench test is carried out on a six-cylinder diesel engine. The parameters of the engine are shown in Table 1. The experiments were conducted in a semi-anechoic chamber with the diesel engine connected to the bench by rigid legs. The power dynamometer is connected to the engine output end through a drive shaft to precisely control its speed and load. The dynamometer (CAC380, Xiangyi Power, Changsha, China) has a rated power of 380 kW, a rated torque of 2300 Nm, and a speed limit of 3790 r/min, which meets the test needs of the diesel engine.

The testing system includes vibration acceleration sensors (PCB 621B40), the signal acquisition front-end (LMS SCADAS Mobile SCM05), and a computer (ThinkPad T530). The vibration frequency of the test engine is usually lower than 10,000 Hz, and the amplitude of vibration acceleration does not exceed 100 g (1 g = 9.8 m/s^2^). The range of the sensor used is 500 g, and the sensitivity is 10 mV/g. The sensor error is less than 10% when the test frequency is less than 18,000 Hz.

**Figure 2:**
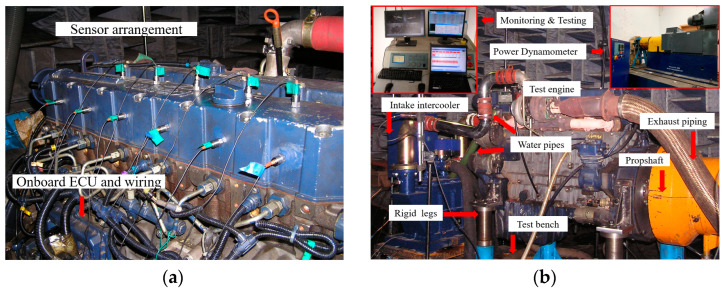
Diesel engine fault simulation experiment: (**a**) The layout positions of the sensors; (**b**) experimental bench.

The cylinder head is closest to the combustion chamber and valve train and contains less noise. The fault simulation experiment mainly collects the vibration signals from the cylinder head, and the sensors are fixed on the cylinder heads of the 1 to 6th cylinders. The location of the sensors and the experimental bench are shown in Figure 2.

The engine structure is complex and fault types are diverse. It is difficult to cover all faults during experiments. Therefore, it is necessary to combine statistical laws to simulate engine faults that occur frequently and are difficult to diagnose. Nahim et al. reviewed and analyzed the main fault types of diesel engines and calculated the probability of various types of faults [31]. The probability of fuel-injection equipment and fuel supply failures, water leaks, and valve and seating failures are higher than other types of failures. Among them, water leakage failure will affect engine cooling and cause the body temperature to be too high. This can be easily monitored through instruments without using complex algorithms for diagnosis. The experiment mainly simulates three kinds of faults (abnormal valve clearance, abnormal rail pressure, and insufficient fuel supply); each fault varies in different degrees. Abnormal valve clearance is designed to simulate changes in valve clearance due to wear and carbon buildup.

Typical causes of common rail system failure are air leaks in the low-pressure line, oil leaks in the high-pressure line, or oil pump failure, all of which will result in a reduction in rail pressure. The injector is installed in the combustion chamber and operates for a long time in an environment of high temperature, high pressure, and gas corrosion. The injector is prone to failure, and the main causes include carbon buildup and wear of the nozzle, which will lead to a reduction in fuel injection. Therefore, abnormal rail pressure and insufficient fuel supply simulate common rail system failure and injector failure, respectively. The valve clearance is changed by a feeler gauge, while the other two faults are adjusted by the ECU. A total of eight types of fault states are included, corresponding to labels 0 to 7. The details are shown in Table 2.

The setting of the sampling frequency must satisfy the Nyquist theorem. If the sampling frequency is chosen improperly, the collected signal will experience aliasing. Through a preliminary experiment conducted before the data collection of the diesel engine, it was found that the vibration frequency of the engine used in the experiment does not exceed 10,000 Hz. According to the Nyquist theorem, the sampling frequency in actual applications should be 2.56 to 4 times the highest frequency of the signal, so that the sampled signal can completely retain the information in the original signal. However, too high a sampling frequency will lead to an excessively large dataset, which is not conducive to subsequent storage and analysis. The sampling frequency is set to 25.6 kHz, and five stable speed conditions of 700 r/min, 1300 r/min, 1600 r/min, 2000 r/min, and 2300 r/min are included in the experiment.

The sample length should be long enough to capture sufficient time series data to effectively identify and analyze the engine’s operating status. Data should also be included for at least one engine operating cycle to cover the possible duration of the failure. The diesel engine used is a four-stroke diesel engine. The crankshaft turns two times in a working cycle. Therefore, the length of a single sample should satisfy (8).

(8) $$l\geq \frac{120\cdot f}{n}$$

where *l* is the length of a single sample, *f* is the sampling frequency (*f* = 25.6 kHz), and *n* is the working speed (units: r/min).

**Figure 3:**
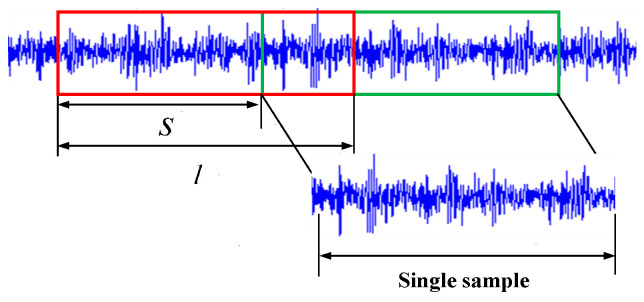
Data augmentation.

When the speed is 2000 r/min, 1536 points are collected in one cycle of the diesel engine. Therefore, the sample length is set to 1600 for convenience of calculation. To enlarge the training dataset, the original time-domain data is intercepted with an overlap rate of 25% (overlap rate = (*l* − *s*)/*l* × 100%). The intercepted samples are represented by boxes of different colors as shown in Figure 3. For the test data, the non-overlap method (overlap rate = 0%) is adopted, which can better simulate the real application scene. The dataset is divided in chronological order. The number of training and testing samples for each fault state at 2000 r/min are 520 and 120, respectively. The length of a sample is the vibration data of the one working cycle of the engine according to Equation (8).

**Figure 4:**
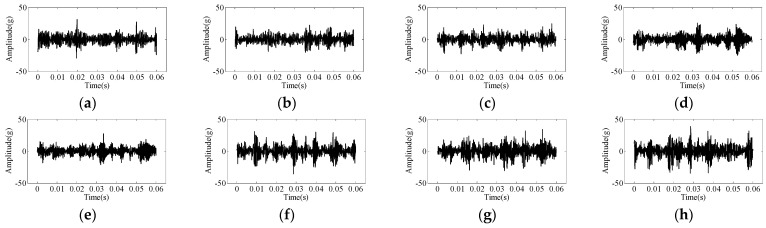
Time domain waveform under different conditions; (**a**) 75% fuel supply, (**b**) 25% fuel supply, (**c**) normal, (**d**) reduced rail pressure by 200 bar, (**e**) reduced rail pressure by 400 bar, (**f**) reduced valve clearance, (**g**) increased valve clearance, (**h**) larger valve clearance.

Figure 4 illustrates the time domain signal waveforms for different working conditions in Table 2. The results show that there is a difference in the waveforms of some types of faults, such as abnormal valve clearance, which is a mechanical fault with an obvious shock waveform. However, the degree of the fault could not be discerned. There is a need to investigate suitable fault diagnosis methods for fast and accurate identification.

## 4. Proposed Method

**Figure 5:**
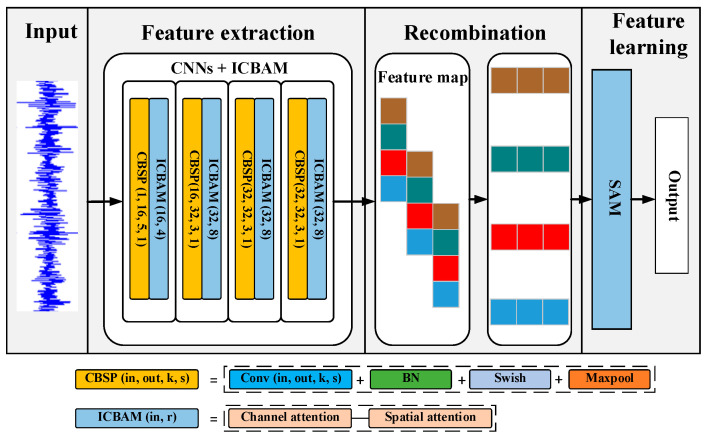
The architecture of the proposed MACNN.

CNN has excellent advantages in feature extraction, and it dramatically speeds up the running by way of local connection and weight sharing. The CNN model has a strong ability to extract local features of data but has limited ability to extract temporal features. For the vibration signal of the engine, its time domain signal usually contains complete working cycle information and has strong time dependence, so it is important to extract its timing characteristics. In time series data, information at different moments may have different importance. The attention mechanism can dynamically adjust the weights so that the model can pay attention to the most important features at each moment, thereby improving the performance of the model. Time series data often have long-range dependencies that may be difficult to capture with traditional models. The attention mechanism can help the model better understand the temporal relationships in time series data, thereby improving the model’s ability to model long-range dependencies. In addition, time series data usually has noise and uncertainty, and the attention mechanism can help the model better cope with these noises and uncertainties, thereby improving the robustness and generalization ability of the model. Therefore, this paper uses CNN as the main body and introduces multiple attention mechanisms to design the end-to-end engine fault diagnosis system MACNN. The structure of MACNN is shown in Figure 5. The diagnosis process includes three steps: feature extraction, feature recombination, and feature learning. Each of them will be described next. All data in Section 4 are from 2000 r/min. The engine vibration signal in the time domain is cut based on engine speed according to Equation (2). The vibration signals are input into the network after normalization.

### 4.1. Feature Extraction

MACNN combines the multi-layer CNN with improved CBAM (ICBAM) to extract features from original time-domain data. The CNN layer alternates with the ICBAM layer. The operating environment of a diesel engine is usually accompanied by a great deal of noise, and the introduction of the attention mechanism makes the model pay more attention to the fault-sensitive information in the signal and ignore the noise part.

The feature extraction phase contains a total of four layers of CNNs. The first convolutional layer uses 16 big convolutional kernels of size 5 × 1 to extract large-scale features. For the next three-layer convolutional layer, 32 small convolutional kernels of size 3 × 1 are used to extract deeper features. After each convolutional layer, a maximum pooling layer (size 2 × 1) is used for down-sampling. Due to the difficulty in training the multi-layer networks, a batch normalization (BN) layer [32] is added between the convolutional layers to further improve the training speed and generalization of the model. The BN layer also reduces the variation between batches and helps the model to better handle noise. The specific structure is shown in Figure 5.

The Swish function [33] is a smooth and continuously derivable function, which is more stable during gradient computation and helps to improve the efficiency of the optimization algorithm and the speed of convergence.

**Figure 6:**
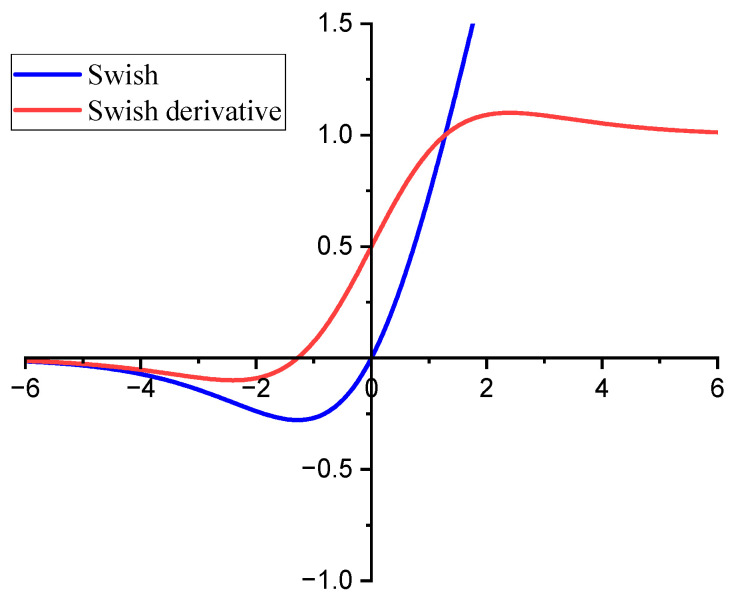
The Swish function and its derivative.

The function expression is Swish(x)=xsigmoid(βx). The Swish function and its derivative curves are shown in Figure 6 (*β* = 1). Swish introduces the Sigmoid function so that the output is non-zero even when the input is in the negative interval. When the input is a positive value, the output of Swish approximates the input (Similar to ReLU). The output does not converge to 0 until the input is a very large negative value, which is equivalent to reducing the effect of negative values and avoiding “Dead ReLU”. Therefore, the output of the convolutional layer is activated using the Swish function. According to [26], *β* is set to 1.

### 4.2. Optimization of ICBAM

The kernel size has a large impact on the performance of CBAM and has different effects on spatial and channel attention, which need to be investigated separately. To determine the kernel size in the channel attention module, we set up five groups of experiments with kernel size 1 × 1, 3 × 1, 5 × 1, 7 × 1, and 11 × 1. The data are divided according to the method described in Section 3, and the time-domain vibration signal is put into MACNN (the CBAM uses channel attention modules only) directly. The cross-entropy is determined as the objective function, and the Adam optimizer is used to update parameters. Each batch contains 256 samples.

The model in this section is built in PyTorch, based on Python 3.8. One NVIDIA GeForce RTX3080 GPU is used for training, and we record accuracy on the test set, as shown in Table 3. Table 3 shows that the kernel size of 7 × 1 in the channel attention module can perform best in extracting features. The result is different from 1 × 1 in the original CBAM. We speculate that the original CBAM is used for two-dimensional data learning tasks such as image processing, which is different from learning tasks for time-series data. Therefore, we will use result 7 × 1 to extract fault features from one-dimensional time-domain data better.

Further, we set up five groups of comparative experiments to determine convolutional kernel size in the spatial attention module, whereby only spatial attention is used in CBAM, and the other experimental conditions are described above. The results are shown in Table 4. The kernel size of 7 × 1 in the spatial attention module can perform best in extracting features. The test results are similar to those of the channel attention, indicating that the 7 × 1 kernel size is more applicable in the processing of engine vibration data.

Another issue of ICBAM that needs to be addressed is the sequential arrangement of the channel and spatial attention modules. Two modules can be placed in a parallel or sequential manner. We set up six groups of experiments, as shown in Table 5. Note that there are two cases (case 1 and case 2) with two modules placed parallelly. Specifically, the final results of case 1 and case 2 are calculated by (9) and (10). 

Case1:

(9) $$P_{1}=(M_{C}(P)⊗M_{S}(P))⊗P$$

where $P_{1}$ is the final result and ⊗ denotes element-wise multiplication.

Case2:

(10) $$P_{2}=M_{C}(P)⊗P+M_{S}(P)⊗P$$

where $P_{2}$ is the final result and ⊗ denotes element-wise multiplication.

The results are shown in Table 5. The channel module and spatial attention module are placed in parallel (case 2) to obtain the best effect in ICBAM. The result differs from the two modules with channel-first order in the original CBAM.

According to the above research, we redesign the ICBAM to extract fault features better from one-dimensional vibration signal data. Specifically, the kernel size in the channel attention module is changed from 1 × 1 to 7 × 1, the kernel size in the spatial attention module remains unchanged at 7 × 1, and the layout of the two modules is changed to a parallel manner (case 2). Compared with the original architecture of CBAM, the ICBAM in MACNN can improve the accuracy from 96.67% to 99.88%, as shown in Table 6. 

The channel reduction ratio is a parameter used to control the number of channels of the attention mechanism, which can significantly reduce the amount of CBAM parameters. The MACNN has a total of four layers of CBSP + ICBAM structure (see Figure 5) in which the first layer of ICBAM has a channel reduction ratio of 0.25 and the other layers have a reduction ratio of 0.125.

### 4.3. Feature Recombination

**Figure 7:**
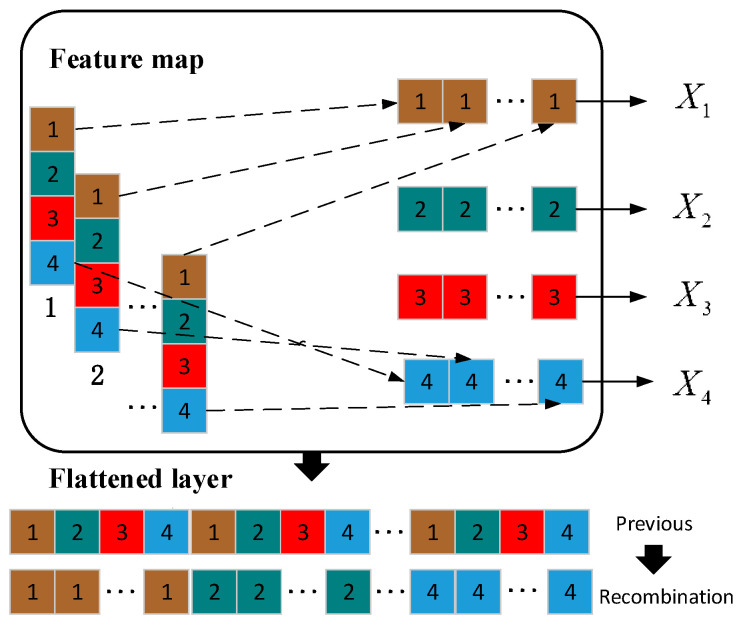
Recombination diagram.

Usually, the multi-dimensional feature map of convolutional output $X=\left\{x_{\_,1},x_{\_,2}\cdot \cdot \cdot x_{\_,d}\right\},\;x_{\_,d}\in {\mathbb{R}}^{l\times 1}$ is flattened into a one-dimensional vector and then input into the fully connected layer to obtain the result in classic CNN. However, this method does not consider the temporal information in the sequence learning tasks. The original sample contains the complete signal of one working cycle of the engine, and the feature maps obtained by the convolution kernel in sliding from front to back still retain certain temporal properties. Unlike picture data, for vibration data in the time domain, temporal features are important. By combining and reconstructing original features to generate new features with more representational capabilities, it can improve the performance and generalization ability of the model. Feature reorganization can help the model capture the relationships and interactions between features, thereby improving the model’s ability to understand the associations between complex data. Therefore, we will recombine the multi-dimensional feature map of convolutional output, as shown in Figure 7. The result of recombination is $X^{\prime }$, as shown in (11).

(11) $$X^{\prime }=\left\{\;x_{1,\_},x_{2,\_}\cdot \cdot \cdot x_{l^{\prime },\_}\;\right\}\;,\;x_{1,\_}\in {\mathbb{R}}^{1\times d}$$

where *l* is the length of the vector $X^{\prime }$. By recombining the feature map, some of the sequence order features in the original signal are preserved, which makes the learning of the SAM layer less difficult and can effectively improve the accuracy. In addition, the attention mechanism can help the model pay more attention to important feature parts when reorganizing features, thereby ensuring that key information is not lost.

### 4.4. Feature Learning

Compared to RNN, SAM is able to capture global information better and solve long-distance dependency problems. And SAM handles sequence feature extraction tasks better than CNN. Therefore, after feature recombination, SAM is used to learn the temporal characteristics of the vibration signal.

Dropout is a regularization method that can randomly discard the output of some neurons during the training process, which helps to prevent the model from overfitting to noise. The dropout layer is set after the SAM to improve the model generalization ability, after which the fully connected layer is connected to output the classification results.

Based on the above derivation, MACNN consists of three steps: (1) Combine CNN with ICBAM to extract the deep features of the original time-domain data. (2) Recombine the multi-dimensional feature map of convolutional output to preserve the sequence order information. (3) Adopt the self-attention mechanism to learn the recombined sequence feature. The procedure of MACNN is shown in Algorithm 1, and the hyper-parameters are shown in Table 7.
**Algorithm 1.** Proposed model**Model: MACNN****Input:** Training set: TRAIN_data and TRAIN_label, test data: TEST_data**Output:** Predicted labels of test data: TEST_label**Training:**1: **for** k =1 . . . K do // forward propagation2:       Calculate the feature map P based on (1), (2), (3).3:       Calculate the feature map after adding attention P2 based on (4), (5), (10).4:       Recombine P2 to obtain recombined sequence based on (11).5:       Use the self-attention to learn the recombined sequence based on (6)~(7).6:       Use Adam optimizer to update parameters. // back propagation7: **end****Testing:** Use TEST_data to predict labels of the test data TEST_label on trained model.

## 5. Result Analysis

### 5.1. Training and Testing

**Figure 8:**
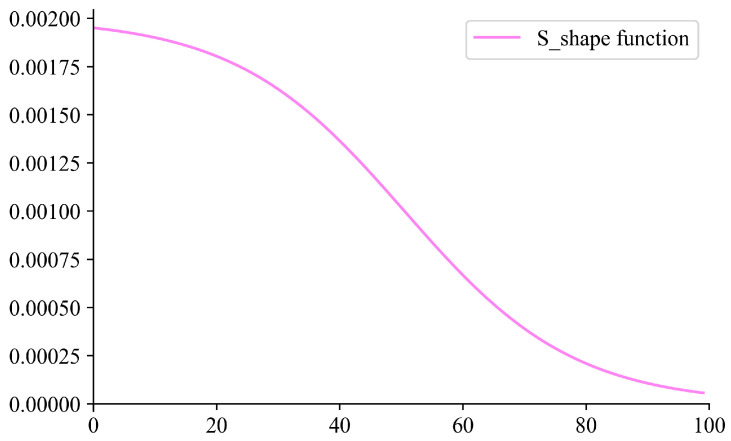
Dynamic learning rate curve.

To improve the convergence performance of the model training, this paper designs a dynamic learning rate parameter so that the optimization method has a higher learning rate in the early stage, and the model can learn the distributional features of the data faster. As the training proceeds, the learning rate gradually decreases and will maintain a lower level at the later stage to maintain convergence. The S-shaped curve just meets the above requirements, so the dynamic learning rate was designed as S-shaped. The function of the dynamic learning rate is shown in (12).

(12) $$lr=lr_{0}/(1+\exp(-k*((e_{\max}-e+1)/W-x_{0})))$$

where *e* stands for epoch $\left(e\in (1,e_{\max})\right)$, $W=(e_{\max}-1)/L$ is the rate of change of the function, *L* denotes the number of epochs needed for the learning rate to reach the plateau stage, $x_{0}=L*(e_{\max}+1)(e_{\max}-1)/2$ denotes the midpoint of the function, and *l_r0_* is the initial learning rate. In this paper, *k* = 0.6, *L* = 12, *lr*_0_ = 0.002, and the curve of *lr* is shown in Figure 8 for an example of iterating 100 epochs.

We trained MACNN on the eight-class fault dataset of the engine, which is described in Section 3. The cross-entropy was determined as the objective function, and the Adam optimizer was used to update parameters. Each training batch contained 256 samples. The learning rate was varied according to (12). At the same time, the accuracy of the test set and the time taken to test 100 samples were recorded.

**Figure 9:**
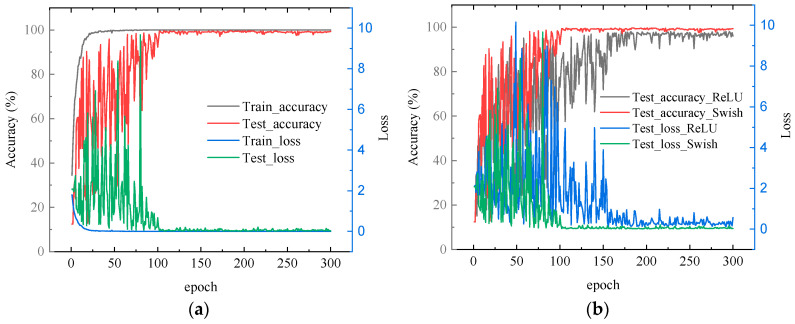
Accuracy and loss curves for training. (**a**) The training process of MACNN; (**b**) improvement of training by the introduction of the swish function.

Figure 9a shows that the MACNN reached the maximum test accuracy (99.88%) after about 100 training steps. It took MACNN 0.35 s to test 100 samples. However, when the speed of the diesel engine was 2000 r/min, the time taken for the diesel engine to work for 100 cycles was 6 s, which is much more than 0.35 s. Therefore, the test results show that MACNN has an excellent effect on accuracy and calculation speed.

It is worth noting that the introduction of the Swish activation function can improve the “Dead ReLU”, which can dramatically improve the training efficiency, as shown in Figure 9b, and shorten the convergence period of the model. Meanwhile, due to the smoother activation curve of Swish, the accuracy is also improved.

### 5.2. Analysis of the MACNN Output

The results in Section 4.1 show that MACNN can reach high accuracy and excellent computation speed. CNN greatly speeds up the running speed through local connection and weight sharing. However, CNN does not consider the order of information for sequence learning tasks. Therefore, we recombined the multi-dimensional feature map to preserve the sequence order. Before that, we introduced the ICBAM to CNN to extract critical information. Finally, we adopted the self-attention mechanism to relate the different positions of the recombined sequence to compute a representation of the sequence. This section will analyze the effectiveness of the ICBAM, recombined method, and the self-attention mechanism on the results.

As shown in Table 8, three models were used for training and testing on the same hardware environment described above. The accuracy and the time taken to test 100 samples of models were compared with the results of MACNN. Specifically, described as follows:

(1) ACNN: This is the model that MACNN lacks ICBAM, which is used to prove the effectiveness of the introduction of ICBAM.
(2) MACNN-noSAM: Same as MACNN, combine the four-layer convolutional network with ICBAM to extract features, and then flatten the recombined sequence features and input them into the fully connected layer.

Compared with ACNN, the test accuracy of MACNN is improved by about 6.0% to 99.88%. Therefore, increasing the time taken (test 100 samples) by 0.2 s is acceptable. Table 8 shows that the self-attention mechanism improves the accuracy from 97.08% to 99.88% due to the self-attention mechanism focusing on the essential parts of the sequence and suppressing unnecessary ones by assigning self-attention weights. The time taken to test 100 samples of MACNN-noSAM is 0.01 s longer than that of MACNN. That is because the length of the input vector of the fully connected layer in MACNN-noSAM is longer than that in MACNN, even if there is an additional self-attention module in MACNN.

### 5.3. Model Evaluation

The datasets under the 2000 r/min condition used by the MACNN model were repartitioned for cross-validation. As described in Section 3, the dataset was divided in chronological order. The first 520 samples were taken as the training set, and the last 120 samples were the testing set in the original division. Four more divisions were obtained by re-dividing the data. The first 120 samples were taken as the testing set and the rest were taken as the training set in the first new division as division 1 in Table 9. By extension, the rest three divisions were obtained. The testing results on the four new divisions are listed in Table 9. Results show that the test accuracies are all higher than 99%, which indicates the proposed MACNN model does not benefit from the particular dataset and has a good robustness.

To verify the validity and generalization ability, another four fault data sets of 700 r/min, 1300 r/min, 1600 r/min, and 2300 r/min were used to train and test the model. We set the length of a single sample as shown in Table 10, and the diagnostic results were also recorded.

Table 10 shows that MACNN can accurately identify different faults at different speeds, and the accuracy at various speeds can reach more than 97%. In addition, the calculation time of the MACNN is positively correlated with the length of the input sample. Because the longer the sample is, the more convolution operations need to be performed in the convolution process. However, it is gratifying that in the current test environment, the calculation speed is much faster than the running speed of the diesel engine. Taking the maximum speed of 2300 r/min as an example, it takes 5.2 s for the diesel engine to work 100 cycles, but it only takes 0.34 s for the model to test 100 samples. Therefore, this model has a good application prospect in terms of diagnostic accuracy and calculation speed.

Table 10 also shows both the recall and precision of the proposed method for each speed case. The results show that the recall and precision results are very close to the accuracy, which is due to the fact that there is no class imbalance in the studied data.

**Figure 10:**
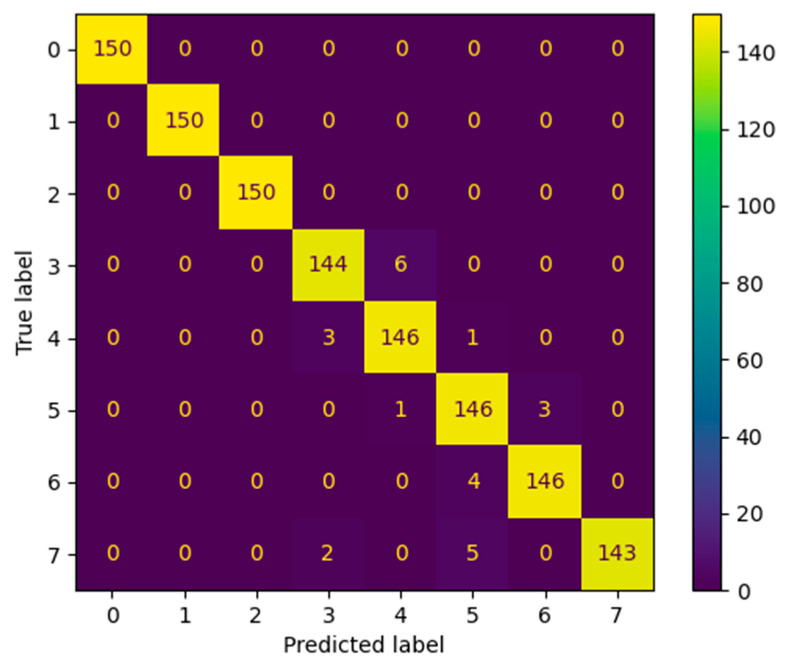
Confusion matrix of MACNN diagnostic results for data at 2300 r/min.

Figure 10 demonstrates the confusion matrix of the proposed method to diagnose the 2300 r/min data. The results show that confusion occurs mainly between classes 3 and 4 and between classes 5, 6, and 7. The accuracy of class 0, class 1, and class 2 is 100%. Table 2 shows that the MACNN made a few errors in distinguishing between different degrees of abnormal rail pressure (classes 3 and 4) and abnormal valve clearance (classes 5, 6, and 7). Different degrees of the same type of fault may lead to similar characteristic changes, making it difficult for the model to distinguish between them. For example, varying fuel supply amounts or valve clearances might cause similar changes in vibration, temperature, or pressure. In practical data, there may be noise or uncertainty, which makes the characteristic changes between the same types of faults less distinct, thus making it challenging for the model to accurately differentiate between them. Notably, no faults were misclassified as normal (class 2), which is important for practical applications.

**Figure 11:**
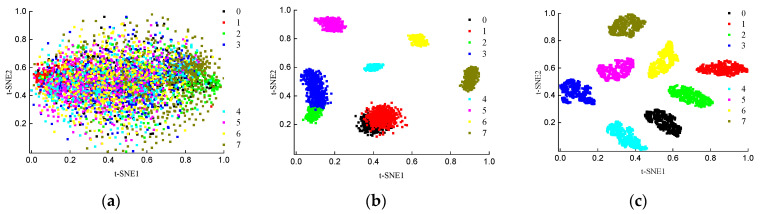
Visualization of t-SNE downscaling. (**a**) Input data, (**b**) CBAM + CNN model, (**c**) recombination features $X^{\prime }$ prior to the SAM layer.

The input layer, the feature layer of CBAM+CNN model and the recombined feature layer of proposed model are downscaled using the t-SNE method respectively, and the 2D visualization results are shown in Figure 11. The distribution of the original signal is very messy and has no obvious clustering characteristics as shown in Figure 11a. The complexity of the components of the engine vibration signal determines that its fault characteristics are difficult to obtain directly through dimensionality reduction. Only four types of faults in the CBAM + CNN model show obvious classification effects in Figure 11b, and the remaining categories are clustered together, indicating that its feature extraction ability is poor and the classification visualization effect is poor. Figure 11c shows that the originally disorganized data presents an obvious clustering effect after ICBAM’s feature extraction as well as feature map reorganization. The results illustrate that the feature extraction of ICBAM + CNN is able to acquire temporal features well.

### 5.4. Comparison with Other Diagnosis Methods

To further evaluate the performance of MACNN, various methods were used to diagnose the eight types of fault data sets established (2000 r/min), mainly including the traditional method based on signal processing and the end-to-end method.

The traditional methods include VMD-KFCM, EEMD-KFCM, and VMD-CNN. The VMD and EEMD decompose the original time-domain signal into intrinsic mode components used to compute feature parameters. The maximum three singular values, kurtosis value, Shannon entropy, root mean square value, time-domain energy, fourth-order cumulant, and multi-scale entropy are extracted to construct 21-dimensional features. The KFCM and CNN are used as classifiers. The CNN is a four-layer convolutional network, and the convolutional kernels’ sizes are all 3 × 1. Traditional methods require manual participation, and the process is cumbersome. The calculation time is much longer than that of MACNN. Therefore, the calculation time of traditional methods is not compared here.

The accuracy is shown in Table 11. The accuracy of EEMD-KFCM is less than 60%. This poor result has much to do with the problem of mode aliasing in the recursive decomposition algorithm, which leads to the poor quality of the final extracted features. VMD extracts the same features, and KFCM can reach an accuracy of 77.29%. For the three-dimensional features (Singular values) extracted by the VMD and the twenty-one-dimensional features, the accuracy of CNN is 36.21% and 80.21%, respectively. The accuracy of VMD-CNN (3D) is low, and we speculate that it is the small number of features that limits the information mining ability of CNNs. The result of VMD-CNN (21D) reflects the powerful feature representation ability of CNN. However, the final diagnostic accuracy of traditional methods is far lower than the proposed method.

As shown in Table 12, various end-to-end methods are used for comparison. In particular, LSTM, BiLSTM, GRU, and BiGRU divide the input sample into 200 data blocks as input. CNN-BiLSTM and CNN-BiGRU adopt two-layer convolutional networks, and BiLSTM and BiGRU have three-layer networks. One-dimensional CNN’s structures and hyperparameters are the same as those of the CNN network in MACNN. The accuracy and the test used time of 100 samples of each model are shown in Table 11.

Table 12 shows that using bidirectional networks (BiLSTM and BiGRU) to consider the information before and after the input position can achieve better results than unidirectional networks (LSTM and BiGRU). And the effect of joint networks (CNN-BiLSTM and CNN-BiGRU) is worse than that of bidirectional networks (BiLSTM and BiGRU). All the above methods based on RNN can achieve end-to-end fault diagnosis. As mentioned above, BiGRU can reach an accuracy of 92.59%. The CNN model can achieve an accuracy of 90.18%. The MACNN proposed can achieve the highest accuracy of 99.88%.

Time complexity is also an important index of an algorithm, and the complexity of each method is analyzed below. The MACNN is used as an example to demonstrate the computation of time complexity. The MACNN contains four main convolutional and attention layers, plus a self-attention layer and a fully connected layer. Since the size of the batch is the same, the effect of the batch is not considered. The time complexity of the one-dimensional convolutional layer is calculated as shown in (13).

(13) $$N_{1\mathrm{DCNN}}=O(L\times K\times I\times O)$$

where *L* is the input sequence length, *K* is the convolution kernel size, and *I* and *O* denote the sizes of input and output channels, respectively.

The complexity of *ICBAM* is summed by the channel and spatial attention as in (14). The complexity of channel and spatial attention is computed similarly to convolutional layers.

(14) $$N_{ICBAM}=O_{channel}+O_{spatial}$$

The time complexity of the self-attention mechanism is given as (15).

(15) $$N_{SAM}=O(H\times L)$$

where *H* is the number of neurons in the hidden layer and *H* = 32 in the model.

The complexity of the fully connected layer is given as (16). *C* is the number of classes.

(16) $$N_{FC}=O(H\times C)$$

The complexity of the *LSTM* layer is calculated via (17), where *l* is the number of layers, *T* is the time step, and 4 represents four gates.

(17) $$N_{LSTM}=O(l\times T\times H\times L\times 4)$$

To calculate the time complexity of each model, a dataset of 1600 r/min with a sample length of 1920 is used as input. The time complexity results for each algorithm are shown in Table 12. The results show that *LSTM* has the lowest time complexity, followed by the other RNN models and finally the CNN-based models. MACNN has the highest complexity but is in the same order of magnitude as CNN-based models. The proposed model significantly improves the accuracy without adding much complexity.

The actual testing time is not only related to the time complexity but also to the parallel computational efficiency of the model. RNN cannot compute in parallel, so it takes 8.12 s to test 100 samples. Considering the actual hardware environment is worse; the running time of the above algorithm will be further increased. It takes only 0.14 s for CNN to test 100 samples. The proposed method takes 0.35 s to test 100 samples, which lays a good foundation for real-time fault diagnosis of diesel engines.

## 6. Conclusions

In this paper, an end-to-end diagnosis system based on MACNN is designed. The proposed MACNN uses CNN as the main body and introduces multiple attention mechanisms to extract features and classify them by self-learning. And the fault simulation experiment of the diesel engine is carried out to collect the vibration signal data of cylinder heads at eight working states. Finally, the results of MACNN verified by the measured vibration signal data show that MACNN can accurately identify different faults at different speeds, and the accuracy at various speeds can reach more than 97%. In the meantime, its fast calculation speed has laid a good foundation for real-time fault diagnosis of diesel engines.

The test data and training data used come from the same working condition in this paper. However, in practical work, most of the operating conditions of mechanical equipment are complex and changeable. Therefore, it is of great practical significance to realize the single-condition data training model to complete the multi-condition fault diagnosis, which will be the future work direction.

## Figures and Tables

**Table 1:** Specifications of the tested diesel engine.

Items	Specifications
Number of cylinders	In-line 6 cylinders
Number of valves/cylinder	4
Displacement	7.14 L
Cylinder diameter/length	108/130 mm
Rated power/speed	220 kW/2300 r/min
Maximum torque/speed range	1250 Nm/1200–1600 r/min

**Table 2:** Fault type.

Label	Fault Type	Fault Degree
0	Insufficient fuel supply (Normal-100%)	75%
1		25%
2	Normal	--
3	Abnormal rail pressure (Normal-1500 bar)	1300 bar
4		1100 bar
5	Abnormal valve clearance (Normal-in 0.30 mm, out 0.50 mm)	(in 0.25, out 0.45)
6		(in 0.35, out 0.55)
7		(in 0.40, out 0.60)

**Table 3:** Kernel size in channel attention module.

Kernel Size	Test Accuracy (%)
1 × 1	93.71
3 × 1	93.72
5 × 1	94.71
7 × 1	95.89
11 × 1	94.08

**Table 4:** Kernel size in spatial attention module.

Kernel Size	Test Accuracy (%)
1 × 1	84.58
3 × 1	95.76
5 × 1	95.09
7 × 1	96.55
11 × 1	90.96

**Table 5:** Combining methods of channel and spatial attention module.

Description	Test Accuracy (%)
channel	95.89
spatial	96.55
channel + spatial	96.67
spatial + channel	96.79
channel and spatial in parallel (case 1)	96.89
channel and spatial in parallel (case 2)	99.88

**Table 6:** Comparison of the original model and improved CBAM.

Description	Test Accuracy (%)
MACNN (with CBAM)	96.67
MACNN (with ICBAM)	99.88

**Table 7:** Specific parameters of the MACNN model.

Layer Name	Output Size	Parametres
Conv1	16	5, stride 1, BN 16, Swish, max pool 2
ICBAM1	16	Channel (7, stride 1) and Spatial (7, stride 1) in parallel (case 2), reduction = 4
Conv2	32	3, stride 1, BN 32, Swish, max pool 2
ICBAM2	32	Channel (7, stride 1) and Spatial (7, stride 1) in parallel (case 2), reduction = 8
Conv3	32	3, stride 1, BN 32, Swish, max pool 2
ICBAM3	32	Channel (7, stride 1) and Spatial (7, stride 1) in parallel (case 2), reduction = 8
Conv4	32	3, stride 1, BN 32, Swish, max pool 2
ICBAM4	32	Channel (7, stride 1) and Spatial (7, stride 1) in parallel (case 2), reduction = 8
Recombine	-	-
Dropout	-	0.5
SAM	-	-
FC	8	Softmax

**Table 8:** Comparison of different models.

Model	Accuracy (%)	Test Used Time (Per 100 Samples)/s
ACNN	93.95	0.15
MACNN-noSAM	97.08	0.36
MACNN	99.88	0.35

**Table 9:** Model evaluation results of different divisions.

Case	Original Division	Division 1	Division 2	Division 3	Division 4
Accuracy (%)	99.88	99.50	99.73	99.81	99.61

**Table 10:** Diagnostic results of different working speeds.

Speed (r/min)	Length (Per Sample)	Accuracy (%)	Recall (%)	Precision (%)	Time/100 Samples (s)
700	4400	98.50	98.51	98.50	0.50
1300	2400	98.96	98.95	98.95	0.40
1600	1920	99.88	99.88	99.89	0.38
2300	1360	98.59	98.57	98.59	0.34

**Table 11:** Comparison with traditional methods.

Method	Accuracy (%)
VMD-KFCM	77.29
EEMD-KFCM	55.42
VMD-CNN(3D)	36.21
VMD-CNN(21D)	80.21
MACNN	99.88

**Table 12:** Comparison with end-to-end methods.

Method	Accuracy (%)	Time Complexity	Time/100 Samples (s)
LSTM	61.04	2 × 10^5^ O	3.67
BiLSTM	88.32	6 × 10^5^ O	7.26
GRU	83.33	5 × 10^5^ O	4.01
BiGRU	92.59	5 × 10^5^ O	8.12
CNN-BiLSTM	80.42	4 × 10^6^ O	7.37
CNN-BiGRU	90.42	4 × 10^6^ O	8.34
1DCNN	90.18	4 × 10^6^ O	0.14
MACNN	99.88	6 × 10^6^ O	0.35

## Data Availability

The data presented in this study are available upon request from the corresponding author.
